# The Role of Oxidative Stress in Suicidal Behaviour Among Bipolar Patients: A Cross-Sectional Study in a Malaysian Sample

**DOI:** 10.3389/fpsyt.2021.698911

**Published:** 2021-11-30

**Authors:** Jiann Lin Loo, Nurul Ain Mohamad Kamal, Jo Aan Goon, Hanafi Ahmad Damanhuri, Jaclyn Ai Chin Tan, Nor Azian Abdul Murad, Shamsul Azhar Shah, Siti Aishah Sulaiman, Shazrul Fazry, Shalisah Sharip, Suriati Mohamed Saini, Geetha Gunasekaran, Thambu Maniam, A. Rahman A. Jamal, Wan Zurinah Wan Ngah, Fatimatul Syahirah Mohd Badli Shah, Lai Fong Chan

**Affiliations:** ^1^Department of Psychiatry, Faculty of Medicine, Universiti Kebangsaan Malaysia Medical Centre, Kuala Lumpur, Malaysia; ^2^Betsi Cadwaladr University Health Board, Wrexham, United Kingdom; ^3^Department of Biochemistry, Faculty of Medicine, Universiti Kebangsaan Malaysia Medical Centre, Kuala Lumpur, Malaysia; ^4^UKM Medical Molecular Biology Institute, Universiti Kebangsaan Malaysia, Kuala Lumpur, Malaysia; ^5^School of Biosciences and Biotechnology, Faculty of Science and Technology, Universiti Kebangsaan Malaysia, Bangi, Malaysia

**Keywords:** suicidal ideation, suicide attempt, suicide, suicidal behaviour, oxidative stress, biomarkers, DNA damage, bipolar disorder

## Abstract

**Background:** Oxidative stress markers are found to be linked with depression and suicide attempts in bipolar disorder (BD), although the role of DNA damage as a marker of suicidal ideation and attempt has yet to be determined. We aim to investigate the association between DNA damage and suicidal behaviour, i.e., suicidal ideation and suicide attempt, among suicidal ideators in BD patients while accounting for clinical and psychosocial risk factors.

**Methods:** A cross-sectional study was conducted in the Universiti Kebangsaan Malaysia Medical Centre on 62 consecutive BD patients diagnosed using the M.I.N.I. Neuropsychiatric Interview and 26 healthy control participants. Socio-demographic and clinical assessments were performed using the Columbia Suicide Severity Rating Scale (C-SSRS) for lifetime suicidal ideation and attempt, Quick Inventory of Depressive Symptomatology (QIDS) for depression severity, Clinical Global Impression for Bipolar Disorder (CGI-BD) for illness severity [both mania (CGI-Mania) and major depressive episode (CGI-MDE)], Social Readjustment Rating Scale (SRRS) for change in life events, and Barratt Impulsiveness Scale (BIS) for behavioural impulsivity. The degree of DNA damage in peripheral blood samples was determined using a standard protocol of comet assay.

**Results:** Multivariable logistic regression revealed higher scores of CGI-MDE as the sole significant factor for lifetime suicidal ideation (OR = 1.937, 95% CI = 1.799–2.076). Although initial bivariate analysis showed a significant association between DNA damage, malondialdehyde (MDA), catalase (CAT), and suicidal behaviour, the findings were not seen in multivariable logistic regression. Bivariate subgroup analysis showed that moderate and severe DNA damage (*p* = 0.032 and *p* = 0.047, respectively) was significantly associated with lifetime suicide attempts among lifetime suicidal ideators. The study is the first to look at the connexion between DNA damage and suicidal risk in bipolar patients. It is limited by the small sample size and lack of information on illicit substance use.

**Conclusions:** More severe DNA damage was significantly associated with lifetime suicide attempts among lifetime suicidal ideators in BD. However, the severity of depression was found to be independently associated with lifetime suicidal ideation *per se* rather than DNA damage in BD. Larger prospective studies are required to ascertain the potential of DNA damage as a biomarker for the transition from suicidal ideation to a suicide attempt.

## Introduction

Suicide is a common endpoint for many individuals with severe psychiatric illnesses. It account for 1.4% of all deaths worldwide and is a significant cause of mortality in bipolar disorder (BD) (1). Recent research has found that 20% of BD patients die by suicide, with 20–60% of them having at least one suicide attempt in their lifetime (2).

The presence of suicidal ideation is a recognised risk factor for more serious suicidal acts (3). However, previous studies have found that not all suicidal ideators progress to attempted or completed suicide. A retrospective chart review by Busch et al. (4) showed that only 39% of patients eventually committed suicide, and most of them were depressed (4). Chan et al. (5) found that 24% of suicidal ideators with unipolar or bipolar depression transitioned to a suicide attempt within a year (5). Therefore, it is clinically pertinent to identify suicide attempters among suicidal ideators given attempted suicide being one of the most robust predictors of eventual suicide (6). Established risk factors for suicidal ideation among BD patients include the severity of hopelessness, irritability directed inwards, and hostility (7). The risk for a suicide attempt is increased in the presence of the following factors: previous suicide attempt, comorbid alcohol abuse, recently been discharged from the hospital, depression, mixed features, and psychotic mania (8).

May and Konskly's meta-analysis has shown that clinical risk factors for suicidal ideators are different from those for suicide attempters; i.e., depression and hopelessness predict suicidal ideas well but not suicide attempts (9). This difference is one of the challenges in the process of predicting suicidal behaviour in clinical settings; i.e., which suicidal ideators will subsequently attempt suicide? Thomas Joiner has introduced the concept of capability for suicide in his interpersonal-psychological theory of suicide (ITPS); i.e., a suicide attempter has developed the capability to overcome the fear of attempting suicide via the habituation to pain, injury, and death (10, 11). This type of ideation-to-action framework has been employed in the process of understanding the progression of a suicidal idea to potentially lethal attempts from the clinical perspective (12). Nevertheless, this model is based solely on clinical factors, and it does not incorporate the measurable biological markers. Also, mental illness appears to play a lesser role in suicide in Asia, while the accessibility to lethal suicide means and acute life stressors appears to be more important as compared with the Western counterpart (13). Some lethal methods for suicide used by the Asian population include charcoal burning, pesticide poisoning, ingestion of native poisonous plants, self-immolation, and jumping (14).

Oxidative stress, a group of measurable biological markers, has been implicated in the pathophysiology of BD. A meta-analysis by Brown et al. (15) stated that increased lipid peroxidation (LP) and nitric oxide (NO) levels, as well as DNA and RNA damage, are commonly found in BD (15). In addition, oxidative stress biomarkers are also shown to be associated with suicide attempts in bipolar depression, which includes NO metabolites (NOx), lipid hydroperoxides, and plasma total antioxidant potential (16). Schiavone et al. have suggested high NOX2-derived oxidative stress may have a role in predicting suicide (17). In the study of Moraes et al. (18), they cannot find any association between oxidative stress and suicidal behaviour, which can be predicted by early life trauma (18). In another study, the NO level and advanced oxidation protein products (AOPP) correlate positively to the intensity of suicidal ideation, and the number of lifetime suicide attempts correlates positively with advanced glycoxidation end products (AGE) and dityrozine (DT), while it correlates negatively with catalase (CAT) (19). A meta-analysis indicates that inflammatory neurotoxicity, nitro-oxidative stress, and lowered neuroprotection explain partly the increased suicidal ideation and attempts among psychiatric patients, which is more in suicide attempters (20).

There is still a knowledge gap with regard to our understanding of the role of oxidative stress markers in more comprehensive risk models of suicidal behaviour incorporating established clinical and psychosocial factors, particularly in terms of differentiating suicide attempters from non-attempters within a high-risk group of BD patients with suicidal ideation, especially in Asian populations. Therefore, the objective of this study is to investigate the association between oxidative stress biomarkers and suicidal behaviour, i.e., suicidal ideation and suicide attempt, among suicidal ideators in BD patients while taking into consideration the interplay of clinical and psychosocial risk factors for suicidal behaviour.

## Methods

### Participants

A cross-sectional study of a total sample of 88 study participants that included 62 consecutive BD inpatients and outpatients and 26 healthy controls from the age group range from 19 to 74 years old was conducted between July 2015 to November 2017 in one of the university hospitals in Malaysia. The diagnosis of BD was confirmed with M.I.N.I. Neuropsychiatric Interview (MINI 7.0) using the *Diagnostic and Statistical Manual of Mental Disorders*, 5th Edition (DSM-5) criteria, in either English (21) or Malay version (22). The patient must be able to communicate in both English or Malay language and give written consent. Exclusion criteria were as follows: clinically too ill to be interviewed, delirium, dementia, mental retardation, anaemia or other blood diseases, inflammatory/rheumatological diseases, metabolic or cancer-related diseases, pregnancy, or refusal to participate in the study. Eligible healthy controls were recruited according to the following inclusion criteria: no lifetime diagnosable psychiatric conditions, no lifetime suicidal ideation, stable medical conditions, and written consent for the study. Exclusion criteria for healthy control included the following: registered staff or student of Universiti Kebangsaan Malaysia Medical Centre (UKMMC), having anaemia or other blood diseases, inflammatory/rheumatological diseases, metabolic, or cancer-related diseases and pregnancy. Age and gender matching for healthy controls was not feasibly performed due to logistic challenges. However, age and gender were accounted for in the multivariable logistic regression analysis. Socio-demographic information, i.e., age, gender, ethnicity, marital status, education level, employment, religion, smoking status, psychotropic medication, and antioxidant use, was collected using a self-reported questionnaire. Columbia Suicide Severity Rating Scale (C-SSRS), an interviewer-rated questionnaire, was used to assess for lifetime suicidal ideation (defined as a “yes” answer in item 3 in the lifetime suicidal ideation section of C-SSRS, i.e., the presence of active suicidal ideation in a lifetime regardless of the intention to carry out) and attempt (defined as a “yes” answer in the column of lifetime actual of attempt in the section of suicidal behaviour in C-SSRS). C-SSRS was suitable for assessment of both suicidal ideation and behaviour given the good convergent and divergent validity for suicidal ideation, i.e., strong correlation to suicide item of Montgomery–Åsberg Depression Rating Scale (MADRS) (*r* = 0.799, *p* < 0.0001; Cohen's d = 2.657) and weak correlations against somatic depression items, i.e., sleep (*r* = 0.021, *p* > 0.05; Cohen's d = 0.042) and hunger (*r* = 0.042, *p* > 0.05, Cohen's d = 0.084). There were high sensitivity and specificity for suicidal behaviour, i.e., 100% sensitivity and specificity for actual suicide attempts (23). The Malay version of C-SSRS was linguistically validated (24).

Disease severity of BD was assessed with clinician-rated Clinical Global Impression for Bipolar Disorder (CGI-BD) and self-rated Quick Inventory of Depressive Symptomatology (QIDS). CGI-BD, an improved version of the original CGI, which was more suitable for the assessment of BD, was used to assess both the severity of manic (CGI-Mania) and major depressive episode (CGI-MDE) (25). A higher CGI-Mania score indicated more severe mania, while a higher CGI-MDE score indicated more severe depression.

The QIDS was a self-reported questionnaire with high internal consistency and concurrent validity for the severity of major depressive episode (MDE) (26). The translation of QIDS into the Malay language was done using the “2 forward, 2 backward” technique. Forward translation from the original English version to the Malay version was performed by a master-level clinical psychologist and a biomedical science research officer with established research experience in the reliability and validity of psychological rating scales. The two translated versions were combined using the best wording based on the consensus, and face validity was assessed by a consultant psychiatrist (A) and a doctorate-level clinical psychologist (C). The combined forward translated Malay version was sent for backward translation into English by two medical undergraduates with training in research including translation of rating scales in psychiatry, and the two backward-translated versions were combined by another consultant psychiatrist (B). Subsequently, face validity of the Malay-translated version was assessed by the two consultant psychiatrists (A and B) and the clinical psychologist (C). Preliminary pilot reliability analysis of QIDS Malay version in 10 patients with an MDE showed good internal consistency, i.e., Cronbach alpha of 0.849. A higher QIDS score indicated more severe depression.

Social Readjustment Rating Scale (SRRS) was a self-rated questionnaire for change in life events. Good concordance (Spearman's rho 0.97–0.91) was demonstrated between the Malaysian and American populations (27). The Malay version translated by Othman (28) had been widely used in the Malaysian population with depression (28–30). A higher SRRS score indicated more changes in life events.

Barratt Impulsiveness Scale (BIS) was used to examine behavioural impulsivity (31). The Malay version of BIS was validated locally in an unpublished dissertation with good internal consistency (Cronbach alpha of 0.77) and test–retest reliability (Spearman rho 0.77) (32). A higher BIS score indicated more behavioural impulsivity.

Ethical approval was obtained from UKMMC Research and Ethics Committee [Approval Number GUP-2014-048].

### Blood Sampling

Peripheral blood measuring 15 ml was sampled within 24 h of the clinical interview in heparinized tubes for the analysis of superoxide dismutase (SOD), glutathione reductase (GPx), CAT activities, and malondialdehyde (MDA) concentrations, as well as for assessment DNA damage using comet assay (CA). The samples were sent to the laboratory for biochemical analysis upon sampling. The samples were initially centrifuged at 1,008 *g* for 10 min at 4**°**C to separate the red blood cells (RBCs) from the plasma. The RBC pellets were washed three times with 0.9% ice-cold NaCl and centrifuged at 1,008 *g* for another 10 min before being stored in the freezer with a temperature of −80**°**C until the time of analysis. Meanwhile, the plasma layer was transferred into Eppendorf tubes for MDA analysis.

#### Superoxide Dismutase Activity Analysis

The activity of SOD was analysed based on the methods by Beyer and Fridovich (33). Blood samples were mixed with an equal volume of distilled water to prepare haemolysates. The haemolysate (20 μl) was added to 1 ml of substrate solution that was made up of 50 mM of phosphate buffer, 0.2 M of l-methionine, 1.72 mM of nitro blue tetrazolium, and 1% Triton X-100. This mixture was then reacted with 10 μl of riboflavin in a brightly illuminated aluminium foil-lined box containing two 20-W Sylvania GroLux fluorescent lamps for 7 min. A control tube was run simultaneously, and the absorbance was measured at 560 nm. One unit of SOD equals the amount of enzyme needed to inhibit the rate of reduction of nitro blue tetrazolium by 50% at 25 °C and pH 7.8.

#### Glutathione Reductase Activity Analysis

The activity of GPx was analysed based on the methods by Paglia and Valentine (34). Firstly, the haemolysates (200 μl) were diluted with 0.8 ml of distilled water and mixed with 1 ml of cyanmethemoglobin reagent. Then it was added to a substrate mixture that was made up of 0.05 M of phosphate buffer, 5 mM of ethylenediamine tetraacetate, 1.125 M of sodium azide, 8.4 mM of reduced nicotine adenine dinucleotide phosphate (NADPH), 10 U/ml of GPx, and 0.15 M of reduced glutathione. Subsequently, 0.1 ml of H_2_O_2_ (2.2 mM) was added into 2.9 ml of the reaction mixture, followed by a change in absorbance for 5 min at 340 nm. A control tube was also run simultaneously. One unit of Px equals the amount of enzyme that was needed to oxidise 1 μmol of NADPH to NADP^+^ per min at 25**°**C and pH 8.0.

#### Catalase Activity Analysis

The activity of CAT was analysed based on the methods by the team of Aebi (35). Initially, the haemolysate was prepared with 50 mM of phosphate buffer. Then 1 ml of H_2_O_2_ (30 mM) was added to 2 ml of enzyme solution to initiate a reaction. The change in absorbance was read against a blank that contained 1 ml of phosphate buffer. One unit of CAT equals the amount of enzyme needed to decompose 1 μmol of H_2_O_2_ per second at 25**°**C at pH 7.0.

#### Malondialdehyde Concentration

The concentration of MDA was measured based on the methods by Pilz et al. (36). Firstly, 25 μl of 1,1,3,3-tetraethoxypropane 95% was dissolved in 100 ml of distilled water to prepare an MDA standard of 1 mM of stock solution. Then 1% sulfuric acid was diluted with the prepared MDA standard to yield final concentrations of 10, 5, 2.5, and 1.25 nmol/ml to obtain the standard curve for the determination of MDA in total.

#### Comet Assay

Assessment of DNA damage was performed using a standard protocol for CA (37, 38). A mixture of 5 μl of whole blood with 70 μl of 0.6% low melting point agarose was prepared at about 37°C and rapidly pipetted onto a fully frosted microscope slide coated with a layer of 80 μl of normal-melting-temperature agarose (0.6%). Coverslip was removed after the solidification process, and the slides were immersed in cold lysing solution (2.5 M of NaCl, 100 mM of Na_2_EDTA·2H_2_O, 10 mM of Tris-HCl, and sodium sarcosinate, adjusted to pH 10.0 with solid NaOH; and the volume was made up of 1 L of dH_2_O) for 1 h in the refrigerator. The slides were then incubated in freshly prepared electrophoresis buffer (60 ml of NaOH and 10 ml of EDTA were made up of 2 L of cold dH_2_O) at 10°C and a depth of 0.25 cm for 20 min. Electrophoresis of DNA was carried out at 1–10°C for 20 min using 25 V with the current adjusted to 300 mA, followed by buffer neutralisation with 0.4 M of Tris (pH 7.5) for two times. Slides were drained, and 30 μl of EtBr (0.2 mg/ml) was added. Slides were placed in a humidified air-tight container in a refrigerator to prevent drying of the gel and analysed immediately after the excess liquid was blotted. Coded slides were examined at ×200 magnification using a fluorescence microscope (AxioCam MRC, Carl Zeiss, Germany) whereby 500 randomly selected non-overlapping cells on each slide were analysed microscopically by categorising cells as undamaged cells without a tail (Grade 0 = CA0), cells with a tiny tail (Grade 1 or mild damage = CA1), cells with a dim tail (Grade 2 or moderate damage = CA2), cells with a clear tail (Grade 3 or severe damage = CA3), and only tail (Grade 4 or maximally damage = CA4). A total damage score for each slide was calculated by multiplying the number of cells assigned to each grade of damage by the numeric value of the grade and summing overall grades, giving a maximum possible score of 2,000, corresponding to 500 cells at grade 4 (CA total) (39).

### Statistical Analysis

The statistical analysis was performed using Statistical Product and Service Solution version 20 (SPSS 20). Statistical differences were analysed using the chi-squared test and Mann–Whitney U-test given the non-normally distributed data. The independent variables with continuous data included age, body mass index (BMI), CGI-Mania, CGI-MDE, QIDS, BIS, SRRS, SOD, GPx, CAT, MDA, CA 0, CA 1, CA 2, CA 3, CA 4, CA 5, and CA Total, when the independent variables with categorical data included gender (female = 1), ethnic (Malay = 1), marital status (non-married = 1), education level (higher educational level = 1), employment (working = 1), religion (Muslim = 1), smoking status (yes = 1), and antioxidant use (yes = 1).

A multivariable binary logistic regression was carried out using lifetime suicidal ideation compared with that without dependent variables.

## Results

The clinical characteristics of BD patients are outlined in Table 1.

**Table 1:** Clinical characteristic of BD patients.

**Characteristics**	***N* (%)**
	**(Total = 62)**
Treatment with lithium	12 (19.35%)
Treatment with atypical antipsychotics	53 (85.48%)
Treatment with anticonvulsants	33 (53.23%)
Bipolar 1 disorder	47 (75.81%)
With mixed features	9 (14.52%)
With chronic medical illness (diabetes mellitus, hypertension, dyslipidaemia)	21 (33.87%)
With lifetime suicidal ideation	27 (43.55%)
With lifetime suicide attempt	16 (25.81%)

*BD, bipolar disorder*.

Table 2 describes the demography, clinical details, and the severity of DNA damage among BD patients with and without suicidal ideation and healthy control. The characteristics of the three groups were similar in terms of age (*p* = 0.114), gender (*p* = 0.133), ethnicity (*p* = 0.661), marital status (*p* = 0.738), religion (*p* = 0.949), and smoking status (*p* = 0.135). Missing data for antioxidant use were 18.2%, and smoking status was 39%.

**Table 2:** Differences in demographics, clinical characteristics, and oxidative stress between case (BD patients with lifetime suicidal ideation) and control (BD patients without lifetime suicidal ideation and healthy control) (*n* = 88).

	**BD patients with lifetime suicidal ideation, *n* = 27**	**Control**, ***n*** **=** **61**		***p*-Value**
		**BD patients without lifetime suicidal ideation, *n* = 35 (IQR or %)**	**Healthy control, *n* = 26 (IQR or %)**	
Age (years), median (IQR)	38 (13)	40 (29)	33 (15)	0.680^*^
BMI, median (IQR)	26.25 (9.58)	25.6 (6.4)	22.64 (5.25)	0.412^*^
Smoking, *n* (%)	8	10	2	0.776^**^
Antioxidant use, *n* (%)	11	21	11	0.785^**^
**Female**, ***n*** **(%)**	**21**	**18**	**14**	0.034**
Malay, *n* (%)	12	20	10	0.818^**^
Non-married, *n* (%)	17	20	14	0.614^**^
Higher education, *n* (%)	19	20	24	0.794^**^
Working, *n* (%)	14	22	21	0.138^**^
Muslim, *n* (%)	13	20	12	0.817^*^
CGI-Mania, median (IQR)	1 (3)	4 (4)	1 (0)	0.921^*^
**CGI-MDE, median (IQR)**	**3 (3)**	**1 (1)**	**1 (0)**	<0.001*
**QIDS, median (IQR)**	**9.5 (10)**	**6 (9)**	**2 (4)**	<0.001*
BIS, median (IQR)	67 (13)	68.5 (13)	66.5 (13)	0.927^*^
SRRS, median (IQR)	300 (241)	333 (424)	263 (214)	0.801^*^
CA 0, median (IQR)	180 (206)	129.5 (200)	366 (113)	0.210^*^
CA 1, median (IQR)	163 (84)	172.5 (88)	173 (113)	0.225^*^
CA 2, median (IQR)	97 (126)	109 (175)	5 (13)	0.073^*^
**CA 3, median (IQR)**	**36 (48)**	**32 (48)**	**1.5 (5)**	0.032*
**CA 4, median (IQR)**	**10 (22)**	**11 (15)**	**0 (2)**	0.038*
CA Total, median (IQR)	524 (121)	525.5 (114)	525.5 (27)	0.351^*^
**MDA, median (IQR)**	**4.59 (1.95)**	**4.49 (1.62)**	**2.23 (0.32)**	0.003*
SOD, median (IQR)	1.58 (0.92)	1.25 (0.8)	1.52 (1.02)	0.623^*^
**CAT, median (IQR)**	**0.45 (0.06)**	**0.44 (0.08)**	**0.38 (0.07)**	0.005*
GPx, median (IQR)	5.1 (3.39)	4.87 (1.67)	5.57 (2.08)	0.568

*IQR, interquartile range; BMI, body mass index; CGI-Mania, Clinical Global Impression for manic episode; CGI-MDE, Clinical Global Impression for major depressive episode; QIDS, Quick Inventory of Depression Symptomatology; BIS, Barratt Impulsiveness Scale; SRRS, Social Readjustment Rating Scale; CA, comet assay; MDA, malondialdehyde; SOD, superoxide dismutase; CAT, catalase; GPx, glutathione reductase; BD, bipolar disorder*.

* *Mann–Whitney U-test*.

** *Chi-squared test. Bolded item, p < 0.05*.

Among the BD patients, 50% (27 subjects) had lifetime suicidal ideation, and 22.22% (12 subjects) had lifetime suicide attempts. A significantly higher proportion of female gender (*p* < 0.034), higher score of CGI-MDE (*p* < 0.001), higher score of QIDS (*p* < 0.001), higher frequency severe DNA damage (CA 3) (*p* < 0.032), maximal DNA damage (CA 4) (*p* < 0.038), higher level of MDA (*p* < 0.005), and higher level of CAT (*p* < 0.003) were seen in the group with lifetime suicidal ideation compared with the healthy control using non-parametric univariate analysis. BIS (*p* = 0.927) and SRRS (*p* = 0.801) were not significantly different between the groups.

After the female gender, CGI-MDE, QIDS, CA 3, CA 4, MDA, and CAT were controlled using multivariable binary logistic regression with ENTER mode (lifetime suicidal ideation as dependent variable), only CGI-MDE was significantly associated with lifetime suicidal idea (Table 3). Higher CGI-MDE was associated with lifetime suicidal ideation among the subjects (OR = 1.937, 95% CI = 1.799–2.076, *p* = 0.017, Cox and Snell *R*^2^ = 0.278; Nagelkerke *R*^2^ = 0.387) as shown in Table 3.

**Table 3:** Effects of demographic factors, clinical factors, and oxidative stress on lifetime suicidal ideation using multivariate binary logistic regression analysis.

**Variable**	**OR**	**SE**	**95% CI**		***p*-Value**	**Wald (df = 1)**
			**Lower**	**Upper**		
Female	0.448	0.643	0.127	1.578	0.211	1.563
**CGI MDE**	**1.937**	**0.277**	**1.126**	**3.331**	**0.017**	**5.705**
QIDS	1.035	0.067	0.908	1.179	0.609	0.261
CA 3	0.989	0.015	0.961	1.018	0.451	0.568
CA 4	1.014	0.030	0.955	1.075	0.655	0.200
MDA	1.356	0.253	0.826	2.224	0.229	1.450
CAT	1,644.754	5.524	0.033	82843092.54	0.180	1.797
**Constant**	**0.002**	**2.579**			**0.017**	**5.692**

*Cox and Snell R^2^ = 0.278; Nagelkerke R^2^ = 0.387. OR, odds ratio; df, degree of freedom; CGI-MDE, Clinical Global Impression for major depressive episode; QIDS, Quick Inventory of Depression Symptomatology; CA, comet Assay; MDA, malondialdehyde; SOD, superoxide dismutase; CAT, catalase. Bolded item, p < 0.05*.

In the subgroup of BD patients with lifetime suicidal ideation (27 subjects), 44.44% (12 subjects) had lifetime suicide attempts. Bivariate subgroup analysis revealed a significantly higher frequency of moderate DNA damage (CA 2) (*p* = 0.032) and severe DNA damage (CA 3) (*p* = 0.047) among BD patients with lifetime suicidal ideation and lifetime suicide attempts as compared with those with lifetime suicidal ideation but never attempted suicide (Table 4). Socio-demographic and clinical independent variables were non-significant factors (*p* ≥ 0.05).

**Table 4:** Differences in the degree of DNA damage (quantified by comet assay) between lifetime suicidal ideators with previous suicide attempt and without previous suicide attempt.

	**Lifetime suicidal ideator with lifetime attempt**		*p*-Value^*^
	**Yes, *n* = 12**	**No, *n* = 15**	
	**Median (IQR)**	**Median (IQR)**	
CGI-MDE	4 (2)	2 (3)	0.059
CGI-Mania	1 (3)	2 (3)	0.516
QIDS	9.5 (7)	8 (13)	0.631
SRRS	314.5 (218)	287 (506)	0.943
BIS	67.5 (13)	67 (16)	0.792
CA 0	147.5 (139)	241 (254)	0.075
CA 1	170 (83)	139 (82)	0.347
**CA 2**	**129 (152)**	**63 (190)**	**0.032**
**CA 3**	**48 (54)**	**31 (39)**	**0.047**
CA 4	10 (17)	10 (24)	0.719
CA Total	511 (117)	533 (122)	0.648

*IQR, interquartile range; CGI-Mania, Clinical Global Impression for manic episode; CGI-MDE, Clinical Global Impression for major depressive episode; QIDS, Quick Inventory of Depression Symptomatology; BIS, Barratt Impulsiveness Scale; SRRS, Social Readjustment Rating Scale; CA, comet assay*.

* *Mann–Whitney U-test. Bolded item, p < 0.05*.

## Discussion

### Suicidal Behaviour, Clinical, and Social Factors

After significant covariates using multivariable binary logistic regression were controlled, clinician-rated depression severity (CGI-MDE) emerged as the independent significant factor associated with lifetime suicidal ideation. Our findings suggest that clinician-rated rather than self-reported severity of depression may be more clinically useful in identifying suicidal ideation among bipolar patients. However, this is in contrast to a study of Oquendo et al. (40), which found that subjective rather than clinician ratings of depression severity predicted future suicidal acts. Oquendo's study included both bipolar and unipolar depression patients, in which we postulate that the latter may have been more forthcoming in terms of self-reported depression severity. Hence, in the process of assessment of suicidal behaviour, the choice of clinician vs. self-reported assessment of depression severity may require consideration of the type of diagnosis, i.e., unipolar or bipolar depression.

In our study, traditional risk factors including gender, marital status, and education level were not found to be associated with suicidal ideation. In addition to that, impulsivity was not found to be different between the groups with suicidal ideation. Despite extensive studies, the role of impulsivity in predicting suicidal behaviour was still not conclusive due to conflicting findings (41). Nevertheless, these findings need to be interpreted with caution given the small sample size of the study.

### Oxidative Stress Biomarkers and Suicidal Behaviour

A higher degree of DNA damage was seen among the group with lifetime suicidal ideation compared with those without suicidal ideation *per se*. However, DNA damage did not remain as a significant factor after controlling for severity of depression in which the latter merged as the sole independent factor associated with suicidal ideation from the multivariable analysis. A possible explanation for this finding may lie in the relationship between the positive correlation of the severity of DNA damage with the severity of BD, which is congruent with the findings of Andreazza et al. (42). Raza et al. (43) proposed the pathophysiology of major psychiatric disorders with oxidative stress, i.e., that DNA damage causes dysfunctional DNA repair that results in abnormal neurotransmission and impaired neuroplasticity on top of dysfunctional energy metabolism in the brain (43). Nevertheless, the causal relationship between DNA damage and suicidal behaviour still requires more in-depth longitudinal study for definitive conclusions. Such findings may herald the potential utility of DNA damage as a biomarker of suicidal behaviour in enhancing the predictive value of existing clinical assessments of suicide risk. In addition, future research is required to elucidate the mechanism(s) underlying the relationship between DNA damage and suicidal behaviour. This may have implications in terms of discovering novel therapeutic targets for suicide prevention.

Lifetime suicide attempts have been found to correlate negatively with the CAT level, i.e., a lower CAT value, although it is not specific to BD patients (19). Nevertheless, the role of CAT in the suicidal behaviour of BD patients may be attributed to its positive correlation with the severity of bipolar depression (44). Although MDA is found to be higher in depression and treatment-resistant BD (44–46), there is limited information on its role in suicidal behaviour.

Interestingly, we demonstrated that the severity of DNA damage was the only significant factor that differentiated suicide attempters from non-attempters among BD patients with lifetime suicidal ideation, albeit in a very small subgroup of patients (*n* = 12). We postulate that in this population of patients who are on the more severe spectrum of suicidal behaviour, oxidative stress levels, i.e., DNA damage, may be more indicative of the extent of bipolar illness pathology in comparison with a cross-sectional measure of depression severity. Future longitudinal studies are warranted to clarify the relationship between DNA damage as a potential oxidative stress biomarker of the transition from suicidal ideation to suicide attempt in BD in view of the cross-sectional nature of our current study, which limits any conclusive inference into the temporal progression of ideation attempt. In addition to a study of Vargas et al. (16), which found an association between oxidative stress markers and suicide attempts in BD, other studies have also shown some evidence of the role of oxidative stress markers besides DNA damage in completed suicides. NOX2 is found to be significantly higher in the cortex of suicide victims (17). In line with that, the NO synthase gene variant has been shown to be more susceptible to violent suicidal behaviour (47). A significant correlation is also found between redox ions and DNA damage in the brain regions of the BD suicide victims (48). Polymorphism in glutathione-*S*-transferases M1 (GSTM1) and T1 (GSTT1), an important gene modifying the body's antioxidant capacity, has also been implicated as a risk factor for higher risk of suicidal behaviour among anxious smokers (49). Nevertheless, most of the studies are not able to provide information about the transition from suicidal ideation to a suicide attempt.

### Lithium, Body Mass Index, Antioxidant, Smoking, and Oxidative Stress

Antioxidant use, lithium use, smoking status, and obesity have an important influence on the oxidative stress biomarkers and might be confounding factors to the severity of DNA damage in our study. Although there were limitations in terms of missing data (i.e., missing data for antioxidant use is 18.2% when smoking status is 39%), there was no significant difference between the two groups for the mentioned four factors. Interpretation of the result needs to take this factor into consideration. Several antioxidant compounds have been found to be a potential agent to be adjunctive therapy to treat BD, including chelated mineral formula, l-tryptophan, magnesium, folic acid, omega-3, and *N*-acetylcysteine (NAC) (50–53). Omega-3 fatty acid supplementation is also shown to reduce surrogate markers of suicidal behaviour, although the study is carried out on small sample size (54). With more understanding about oxidative stress in BD patients, a specific agent may be potentially developed to target and treat suicidal behaviour, in conjunction with the traditional treatment regime.

Only a small number of the patients in this study were on lithium (16.67%, i.e., nine of the BD patients), which is an established effective suicide-reduction agent in BD patients. Lithium has been shown to reduce LP and hence reducing oxidative stress in BD patients (55). The anti-inflammatory effect of lithium is also being proposed to involve the inhibition of glycogen synthase kinase-3 (GSK3) pathway, which is linked with the suicidal endophenotypes of aggression, impulsivity, and depression traits (56).

The median BMI of the group of lifetime suicidal ideation was touching the line of overweight, i.e., BMI 25. It is important to note that obesity increases the risk of suicidal behaviour (57) and also has a significant association with a higher level of oxidative stress (58, 59). Hence, obesity might be a potential target of intervention for the prevention of suicidal behaviour.

Vargas et al. have found out that smoking behaviour has an effect on both oxidative stress and suicide attempts (16). Significantly higher levels of NOx, fibrinogen, advanced oxidative protein product, and lower levels of TRAP are seen among depressed smokers compared with non-smoker non-depressed people, and also more frequent suicide attempts (60). This might imply that smoking cessation may be a potential target to reduce both suicidal behaviour and oxidative stress.

### Strength

To the best of our knowledge, DNA damage in bipolar patients connected with redox state and suicide risk has not been described yet. As CA is a relatively cost-effective biochemical analysis, the findings of this study highlight the possibility of using basic oxidative stress analysis to elucidate suicidal behaviour further.

### Limitations

Cross-sectional study design limits the interpretation of the causal relationship of this association, i.e., the progression from suicidal ideation to attempt cannot be demonstrated. The small sample size subjects this study to potential type 2 error. Multiple testing increases the possibility of type 1 error. Another limitation is that the information on illicit substance use is not captured systematically using specific measurement tools, although our clinical interview only found that one of the BD patients has a significant substance use issue. In addition, the Malay versions of QIDS and SSRS do not have full-scale validation. The pharmacological treatments reported in Table 1 (i.e., lithium, atypical antipsychotics, and anticonvulsants) were not considered as variables in the performed logistic regression due to the imbalance between medicated BD patients (overrepresented) and non-medicated healthy controls (underrepresented). This is a limitation of our study, as the type of pharmacological treatment is an important confounding variable. Recommendations for future research to address such a study limitation include adequately powered sample sizes and stratification for study participants with and without pharmacological treatments.

## Conclusion

Our study findings suggest that DNA damage may be a potential candidate as a biomarker of suicidal behaviour, in particular differentiating suicide attempters from non-attempters among suicidal ideators in BD. However, the cross-sectional nature and relatively small sample size of our study are limitations that preclude any definitive conclusions with regard to the temporal and causal relationship between DNA damage and suicidal behaviour in BD. Larger prospective studies are necessary to elucidate the mechanism of DNA damage in the transition from ideation into suicide attempt, which in turn would bear clinical implications to the early detection and prevention of the progression of suicidal behaviour in BD.

## Data Availability Statement

The raw data supporting the conclusions of this article will be made available by the authors upon reasonable request.

## Ethics Statement

The studies involving human participants were reviewed and approved by Universiti Kebangsaan Malaysia Medical Centre Research and Ethics Committee. The patients/participants provided their written informed consent to participate in this study.

## Author Contributions

JL, NM, JG, HA, NA, SShah, SF, SShar, SM, TM, AA, WW, and LC: study conception and design. JL, NM, JT, SSu, SShar, GG, FM, and LC: conducting the research. JL, NM, JT, SShah, SSu, and LC: drafting of the manuscript. All authors: approval of the final manuscript.

## Funding

This research was funded by Universiti Kebangsaan Malaysia (Project Code UKM GUP-2014-048).

## Conflict of Interest

The authors declare that the research was conducted in the absence of any commercial or financial relationships that could be construed as a potential conflict of interest.

## Publisher's Note

All claims expressed in this article are solely those of the authors and do not necessarily represent those of their affiliated organizations, or those of the publisher, the editors and the reviewers. Any product that may be evaluated in this article, or claim that may be made by its manufacturer, is not guaranteed or endorsed by the publisher.

## References

[1] World Health Organisation. Suicide Data. (2015). Available online at: http://www.who.int/mental_health/prevention/suicide/suicideprevent/en (accessed January 14, 2019).

[2] Dome P Rihmer Z Gonda X. Suicide risk in bipolar disorder: a brief review. Medicina. (2019) 55:403. 10.3390/medicina55080403 31344941 PMC6723289

[3] Mendez-Bustos P de Leon-Martinez V Miret M Baca-Garcia E Lopez-Castroman J. Suicide reattempters: a systematic review. Harv Rev Psychiatry. (2013) 21:281–95. 10.1097/HRP.0000000000000001 24201820

[4] Busch KA Fawcett J Jacobs DG. Clinical correlates of inpatient suicide. J Clin Psychiatry. (2003) 64:14–9. 10.4088/JCP.v64n0105 12590618

[5] Chan LF Shamsul AS Maniam T. Are predictors of future suicide attempts and the transition from suicidal ideation to suicide attempts shared or distinct: a 12-month prospective study among patients with depressive disorders. Psychiatry Res. (2014) 220:867–73. 10.1016/j.psychres.2014.08.055 25240940

[6] Michael Bostwick J Pabbati C Geske JR McKean AJ. Suicide attempt as a risk factor for completed suicide: even more lethal than we knew. Am J Psychiatry. (2016) 173:1094–100. 10.1176/appi.ajp.2016.15070854 27523496 PMC5510596

[7] Umamaheswari V Avasthi A Grover S. Risk factors for suicidal ideations in patients with bipolar disorder. Bipolar Disord. (2014) 16:642–51. 10.1111/bdi.12179 24467510

[8] Simpson SG Jamison KR. The risk of suicide in patients with bipolar disorders. J Clin Psychiatry. (1999) 60:53–56. 10073388

[9] May AM Klonsky ED. What distinguishes suicide attempters from suicide ideators? a meta-analysis of potential factors. Clin Psychol Sci Pract. (2016) 23:5–20. 10.1037/h0101735

[10] Joiner T. Why People Die by Suicide. Cambridge, MA: Harvard University Press (2005).

[11] Van Orden KA Witte TK Cukrowicz KC Braithwaite SR Selby EA Joiner TE. The interpersonal theory of suicide. Psychol Rev. (2010) 117:575–600. 10.1037/a0018697 20438238 PMC3130348

[12] Klonsky ED Qiu T Saffer BY. Recent advances in differentiating suicide attempters from suicide ideators. Curr Opin Psychiatry. (2017) 30:15–20. 10.1097/YCO.0000000000000294 27798483

[13] Chen Y-Y Chien-Chang Wu K Yousuf S Yip PSF. Suicide in asia: opportunities and challenges. Epidemiol Rev. (2012) 34:129–44. 10.1093/epirev/mxr025 22158651

[14] Wu KC-C Chen Y-Y Yip PSF. Suicide methods in Asia: implications in suicide prevention. Int J Environ Res Public Health. (2012) 9:1135–58. 10.3390/ijerph9041135 22690187 PMC3366604

[15] Brown NC Andreazza AC Young LT. An updated meta-analysis of oxidative stress markers in bipolar disorder. Psychiatry Res. (2014) 218:61–8. 10.1016/j.psychres.2014.04.005 24794031

[16] Vargas HO Nunes SO Pizzo de Castro M Bortolasci CC Sabbatini Barbosa D Kaminami Morimoto H. Oxidative stress and lowered total antioxidant status are associated with a history of suicide attempts. J Affect Disord. (2013) 150:923–30. 10.1016/j.jad.2013.05.016 23856278

[17] Schiavone S Neri M Mhillaj E Morgese MG Cantatore S Bove M. The NADPH oxidase NOX2 as a novel biomarker for suicidality: evidence from human post mortem brain samples. Transl Psychiatry. (2016) 6:e813. 10.1038/tp.2016.76 27187235 PMC5070044

[18] Moraes JB Maes M Roomruangwong C Bonifacio KL Barbosa DS Vargas HO. In major affective disorders, early life trauma predict increased nitro-oxidative stress, lipid peroxidation and protein oxidation and recurrence of major affective disorders, suicidal behaviors and a lowered quality of life. Metab Brain Dis. (2018) 33:1081–96. 10.1007/s11011-018-0209-3 29542039

[19] Koweszko T Gierus J Zalewska A Maciejczyk M Waszkiewicz N Szulc A. The relationship between suicide and oxidative stress in a group of psychiatric inpatients. J Clin Med. (2020) 9:3462. 10.3390/jcm9113462 33126414 PMC7693103

[20] Vasupanrajit A Jirakran K Tunvirachaisakul C Solmi M Maes M. Inflammation and nitro-oxidative stress in current suicidal attempts and current suicidal ideation: a systematic review and meta-analysis. medRxiv. (2021). 10.20944/preprints202109.0159.v1 34997194

[21] Sheehan DV Lecrubier Y Sheehan KH Amorim P Janavs J Weiller E. The mini-international neuropsychiatric interview (M.I.N.I.): the development and validation of a structured diagnostic psychiatric interview for DSM-IV and ICD-10. J Clin Psychiatry. (1998) 59:22–33. 9881538

[22] Mukhtar F Bakar AKA Junus MM Awaludin A Aziz SA Midin M. A preliminary study on the specificity and sensitivity values and inter-rater reliability of mini international neuropsychiatric interview (MINI) in Malaysia. ASEAN J Psychiatry. (2012) 13:157–64.

[23] Posner K Brown GK Stanley B Brent DA Yershova KV Oquendo MA. The Columbia-suicide severity rating scale: initial validity and internal consistency findings from three multisite studies with adolescents and adults. Am J Psychiatry. (2011) 168:1266–77. 10.1176/appi.ajp.2011.10111704 22193671 PMC3893686

[24] The Columbia Lighthouse Project. The Columbia Scale (C-SSRS) Translation. (2016). Available online at: http://cssrs.columbia.edu/the-columbia-scale-c-ssrs/translations/ (accessed May 30, 2017*)*.

[25] Spearing MK Post RM Leverich GS Brandt D Nolen W. Modification of the clinical global impressions (CGI) scale for use in bipolar illness (BP): the CGI-BP. Psychiatry Res. (1997) 73:159–71. 10.1016/S0165-1781(97)00123-6 9481807

[26] Trivedi MH Rush AJ Ibrahim HM Carmody TJ Biggs MM Suppes T. The inventory of depressive symptomatology, clinician rating (IDS-C) and self-report (IDS-SR), and the quick inventory of depressive symptomatology, clinician rating (QIDS-C) and self-report (QIDS-SR) in public sector patients with mood disorders: a psychometric evaluation. Psychol Med. (2004) 34:73–82. 10.1017/S0033291703001107 14971628

[27] Woon T-H Masuda M Wagner NN Holmes TH. The social readjustment rating scale: a cross-cultural study of Malaysians and Americans. J Cross Cult Psychol. (1971) 2:373–86. 10.1177/002202217100200407

[28] Othman AH. Life changes stress of Malay students in the United States. Jurnal Psikologi Malaysia. (1986) 2:129–39.

[29] Chan LF Maniam T Shamsul AS. Suicide attempts among depressed inpatients with depressive disorder in a Malaysian sample. psychosocial and clinical risk factors. Crisis. (2011) 32:283–7. 10.1027/0227-5910/a000088 21940256

[30] Chan LF Shah SA Maniam T. Predictors of suicidal ideation among depressed inpatients in a Malaysian sample. Suicidol Online. (2012) 3:33–41.

[31] Patton JH Stanford MS Barratt ES. Factor structure of the barratt impulsiveness scale. J Clin Psychol. (1995) 51:768–74. 10.1002/1097-4679(199511)51:6<768::aid-jclp2270510607>3.0.co;2-1 8778124

[32] Ahmad Shahabuddin F. Validation study of the malay version of the Barratt Impulsivity Scale (BIS)-11 among psychiatric population (Doctor in Psychiatry's Dissertation). Universiti Kebangsaan Malaysia, Kuala Lumpur, Malaysia (2017).

[33] Beyer WF Fridovich I. Assaying for superoxide dismutase activity: some large consequences of minor changes in conditions. Anal Biochem. (1987) 161:559–66. 10.1016/0003-2697(87)90489-1 3034103

[34] Paglia DE Valentine WN. Studies on the quantitative and qualitative characterization of erythrocyte glutathione peroxidase. J Lab Clin Med. (1967) 70:158–69. 6066618

[35] AEBI H WYSS SR SCHERZ B SKVARIL F. Heterogeneity of erythrocyte catalase II. Eur J Biochemist. (1974) 48:137–45. 10.1111/j.1432-1033.1974.tb03751.x 4141308

[36] Pilz J Meineke I Gleiter CH. Measurement of free and bound malondialdehyde in plasma by high-performance liquid chromatography as the 2,4-dinitrophenylhydrazine derivative. J Chromatogr B Biomed Sci Appl. (2000) 742:315–25. 10.1016/S0378-4347(00)00174-2 10901136

[37] Singh NP McCoy MT Tice RR Schneider EL. A simple technique for quantitation of low levels of DNA damage in individual cells. Exp Cell Res. (1988) 175:184–91. 10.1016/0014-4827(88)90265-0 3345800

[38] Tice RR Agurell E Anderson D Burlinson B Hartmann A Kobayashi H. Single cell gel/comet assay: guidelines for in vitro and in vivo genetic toxicology testing. Environ Mol Mutagen. (2000) 35:206–21. 10.1002/(sici)1098-2280(2000)35:3 <206::aid-em8>3.0.co;2-j 10737956

[39] Heaton PR Ransley R Charlton CJ Mann SJ Stevenson J Smith BH. Application of single-cell gel electrophoresis (comet) assay for assessing levels of DNA damage in canine and feline leukocytes. J Nutr. (2002) 132:S1598–603. 10.1093/jn/132.6.1598S 12042468

[40] Oquendo MA Galfalvy H Russo S Ellis SP Grunebaum MF Burke A. Prospective study of clinical predictors of suicidal acts after a major depressive episode in patients with major depressive disorder or bipolar disorder. Am J Psychiatry. (2004) 161:1433–41. 10.1176/appi.ajp.161.8.1433 15285970

[41] Sloan ME Iskric A Low NC. The treatment of bipolar patients with elevated impulsivity and suicide risk. J Psychiatry Neurosci. (2014) 39:E34–35. 10.1503/jpn.130274 24963644 PMC4074240

[42] Andreazza AC Frey BN Erdtmann B Salvador M Rombaldi F Santin A. DNA damage in bipolar disorder. Psychiatry Res. (2007) 153:27–32. 10.1016/j.psychres.2006.03.025 17582509

[43] Raza MU Tufan T Wang Y Hill C Zhu MY. DNA damage in major psychiatric diseases. Neurotox Res. (2016) 30:251–67. 10.1007/s12640-016-9621-9 27126805 PMC4947450

[44] Lv Q Hu Q Zhang W Huang X Zhu M Geng R. Disturbance of oxidative stress parameters in treatment-resistant bipolar disorder and their association with electroconvulsive therapy response. Int J Neuropsychopharmacol. (2020) 23:207–16. 10.1093/ijnp/pyaa003 31967315 PMC7177162

[45] Liu T Zhong S Liao X Chen J He T Lai S. A meta-analysis of oxidative stress markers in depression. PLoS ONE. (2015) 10:e0138904. 10.1371/journal.pone.0138904 26445247 PMC4596519

[46] Islam MR Islam MR Ahmed I Moktadir AA Nahar Z Islam MS. Elevated serum levels of malondialdehyde and cortisol are associated with major depressive disorder: a case-control study. SAGE Open Med. (2018) 6. 10.1177/2050312118773953 29770218 PMC5946642

[47] Oliveira J Debnath M Etain B Bennabi M Hamdani N Lajnef M. Violent suicidal behaviour in bipolar disorder is associated with nitric oxide synthase 3 gene polymorphism. Acta Psychiatr Scand. (2015) 132:218–25. 10.1111/acps.12433 25939888

[48] Mustak MS Hegde ML Dinesh A Britton GB Berrocal R Rao KS. Evidence of altered DNA integrity in the brain regions of suicidal victims of Bipolar Depression. Indian J Psychiatry. (2010) 52:220–8. 10.4103/0019-5545.70974 21180406 PMC2990821

[49] Odebrecht Vargas Nunes S Pizzo de Castro MR Ehara Watanabe MA Guembarovski RL Vargas HO Reiche EMV. Genetic polymorphisms in glutathione-S-transferases are associated with anxiety and mood disorders in nicotine dependence. Psychiatric Genetics. (2014) 24:87–93. 10.1097/YPG.0000000000000023 24637631 PMC4004636

[50] Magalhaes PV Dean OM Bush AI Copolov DL Malhi GS Kohlmann K. N-acetylcysteine for major depressive episodes in bipolar disorder. Rev Bras Psiquiatr. (2011) 33:374–8. 10.1590/S1516-44462011000400011 22189927

[51] Sarris J Mischoulon D Schweitzer I. Adjunctive nutraceuticals with standard pharmacotherapies in bipolar disorder: a systematic review of clinical trials. Bipolar Disord. (2011) 13:454–65. 10.1111/j.1399-5618.2011.00945.x 22017215

[52] Sarris J Mischoulon D Schweitzer I. Omega-3 for bipolar disorder: meta-analyses of use in mania and bipolar depression. J Clin Psychiatry. (2012) 73:81–6. 10.4088/JCP.10r06710 21903025

[53] Berk M Dean OM Cotton SM Jeavons S Tanious M Kohlmann K. The efficacy of adjunctive N-acetylcysteine in major depressive disorder: a double-blind, randomized, placebo-controlled trial. J Clin Psychiatry. (2014) 75:628–36. 10.4088/JCP.13m08454 25004186

[54] Hallahan B Hibbeln JR Davis JM Garland MR. Omega-3 fatty acid supplementation in patients with recurrent self-harm. single-centre double-blind randomised controlled trial. Br J Psychiatry. (2007) 190:118–22. 10.1192/bjp.bp.106.022707 17267927

[55] Banerjee U Dasgupta A Rout JK. Effects of lithium therapy on Na+-K+-ATPase activity and lipid peroxidation in bipolar disorder. Prog Neuro-Psychopharmacol Biol Psychiatry. (2012) 37:56–61. 10.1016/j.pnpbp.2011.12.006 22230647

[56] Beurel E Jope RS. Inflammation and lithium: clues to mechanisms contributing to suicide-linked traits. Transl Psychiatry. (2014) 4:e488. 10.1038/tp.2014.129 25514751 PMC4270310

[57] Wagner B Klinitzke G Brähler E Kersting A. EXTREME OBESITY IS ASSOCIATED WITH SUICIDAL BEHAVIOR AND SUICIDE ATTEMPTS IN ADULTS: RESULTS OF A POPULATION-BASED REPRESENTATIVE SAMPLE. Depress Anxiety. (2013) 30:975–81. 10.1002/da.22105 23576272

[58] Furukawa S Fujita T Shimabukuro M Iwaki M Yamada Y Nakajima Y. Increased oxidative stress in obesity and its impact on metabolic syndrome. J Clin Invest. (2004) 114:1752–61. 10.1172/JCI21625 15599400 PMC535065

[59] Fernandez-Sanchez A Madrigal-Santillan E Bautista M Esquivel-Soto J Morales-Gonzalez A Esquivel-Chirino C. Inflammation, oxidative stress, and obesity. Int J Mol Sci. (2011) 12:3117–32. 10.3390/ijms12053117 21686173 PMC3116179

[60] Vargas HO Nunes SOV de Castro MRP Vargas MM Barbosa DS Bortolasci CC. Oxidative stress and inflammatory markers are associated with depression and nicotine dependence. Neurosci Lett. (2013) 544:136–40. 10.1016/j.neulet.2013.03.059 23583694

